# Association between serum 25-hydroxyvitamin D and fasting blood glucose in osteoporosis patients

**DOI:** 10.1038/s41598-023-45504-6

**Published:** 2023-11-01

**Authors:** Yao-wei Ye, Ke Lu, Yi Yin, Xu-feng Yang, Si-ming Xu, Min-zhe Xu, Qin Shi, Ya-qin Gong

**Affiliations:** 1 Department of Orthopedics, The First People’s Hospital of Kunshan, Gusu School, Nanjing Medical University, Suzhou, 215300 Jiangsu China; 2 Department of Orthopedics, Affiliated Kunshan Hospital of Jiangsu University, Suzhou, 215300 Jiangsu China; 3 Department of Orthopedics, The First Affiliated Hospital of Soochow University, Orthopedic Institute of Soochow University, Suzhou, 215031 Jiangsu China; 4 Information Department, Affiliated Kunshan Hospital of Jiangsu University, Suzhou, 215300 Jiangsu China

**Keywords:** Endocrinology, Health care, Pathogenesis, Risk factors

## Abstract

Osteoporosis (OP) is often associated with other complications, such as impaired glucose homeostasis. Vitamin D deficiency is common and has been linked to bone metabolism and the regulation of blood sugar levels. The aim of this study was to evaluate the independent relationship between serum 25-hydroxyvitamin D (25[OH]D) and fasting blood glucose levels (FBG) in a group of patients diagnosed with OP. This is a retrospective cross-sectional study from a prospectively collected database at our tertiary referral center. Consecutive 2084 OP patients who were hospitalization were finally analyzed in this study. FBG is the dependent variable, serum 25(OH)D level of OP patients is exposure variable of this study. There was a linear significantly negative association between serum 25(OH)D and FBG (β, − 0.02; 95% CI − 0.03 to − 0.01; *P* = 0.0011) in the fully adjusted models. Specifically, when serum 25(OH)D level was less than 23.39 ng/mL, FBG decreased by 0.04 mmol/L for every 1 ng/mL increase of serum 25(OH)D level. When serum 25(OH)D was greater than 23.39 ng/ mL, the negative association was insignificant (*P* = 0.9616). If the association is confirmed, the clinical management of blood glucose in OP patients with serum 25(OH)D deficiency has instructive implications.

## Introduction

Hip fractures that occur in Asia are forecast to account for half of all global hip fractures by 2050^1^. Osteoporosis (OP) is the most common bone disorder in the world, resulting in reduced bone strength, decreases in bone mass, and microarchitectural bone deterioration^2^. Type 2 diabetes mellitus (T2DM) and OP are both highly prevalent chronic diseases, and the association between the two is an area of active research. Diabetes is a group of metabolic diseases characterized by hyperglycemia resulting from defects in insulin secretion, insulin action, or both. T2DM, which accounts for 90–95% of those with diabetes previously referred to as non–insulin-dependent diabetes, T2DM, or adult-onset diabetes, encompasses individuals who have insulin resistance and usually have relative (rather than absolute) insulin deficiency^3^. Diabetes increases rapidly in the world, especially in Asia. In Asian countries, the predicted prevalence of diabetes by the year 2030 is more than double-rates that in 2000.

Both T2DM and OP are affected by aging and quite often coexist. Furthermore, the fracture risk in patients with T2DM is increased^4^. In T2DM hyperinsulinemia, insulin resistance and increased body weight may result in an increase of bone mass, however, accumulation of advanced glycation end products within the bone collagen driven by glucotoxicity may increase the cortical porosity^5^.It is generally agreed that vitamin D is responsible for maintaining normal levels of serum Ca and P. The insulin resistance was more in vitamin D deficiency state^6^. Previous research show that 25-hydroxyvitamin D (25[OH]D) concentrations are negatively correlated with insulin resistance and bone turnover^7^. Elevated blood sugar raises the risk of OP in patients with T2DM^8^. Taking vitamin D can prevent OP or improve outcomes^9^. However, little evidence was found to exploring the association between blood glucose levels and 25(OH)D in patients with OP. Therefore, we conducted a study and hypothesized that fasting glucose is negatively correlated with 25(OH)D in the OP population.

## Materials and methods

### Study design and population

**Figure 1:**
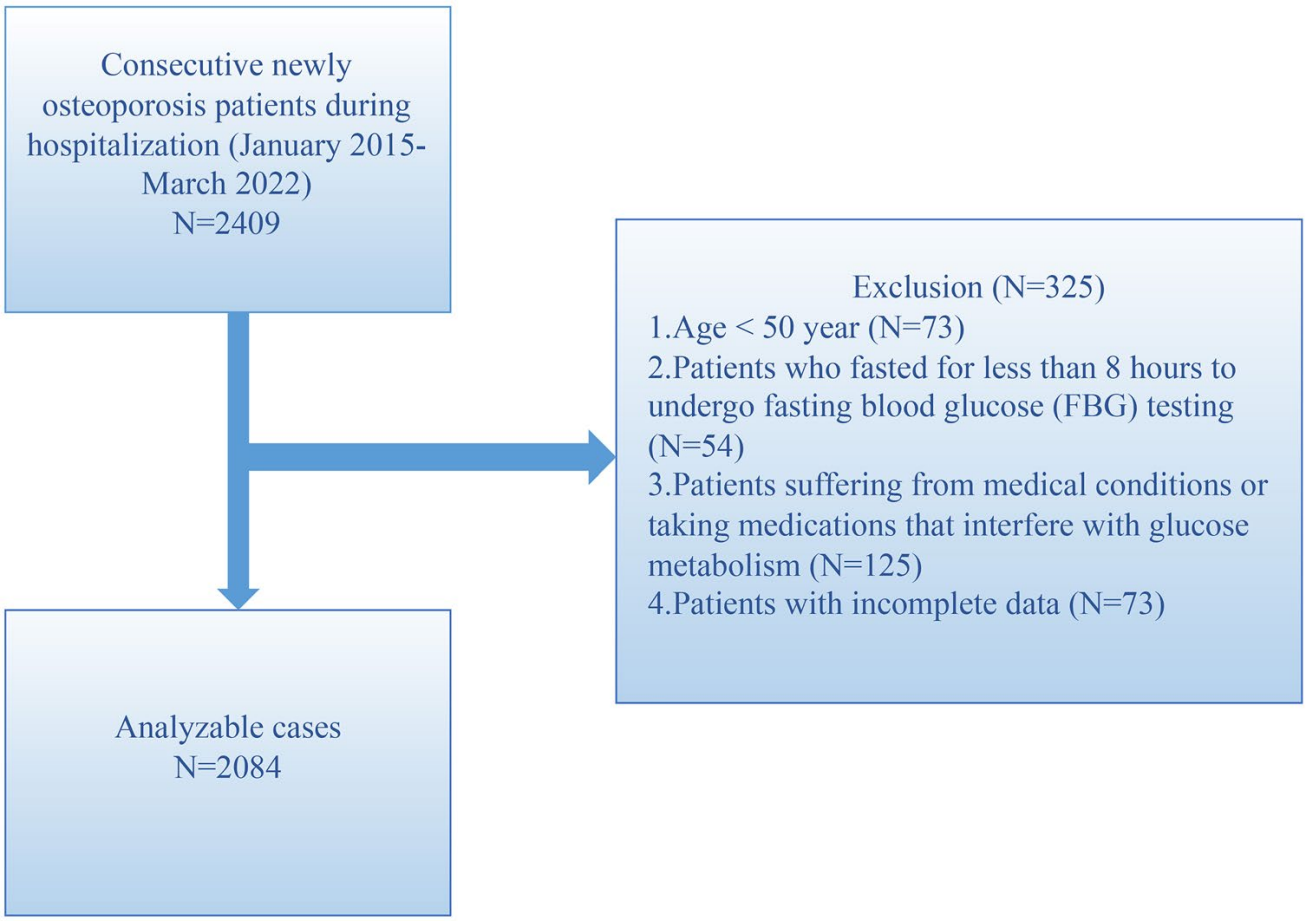
A schematic diagram of the study design. FBG, fast blood glucose.

This is a retrospective cross-sectional study from a prospectively collected database (January 2015–March 2022) at our tertiary referral center. A total of 2409 consecutive patients with newly diagnosed OP and who were hospitalized were included in the study. The study population included OP patients older than 50 years who visited the clinic between 2015 and 2022. OP is diagnosed based on the presence of fragility fractures in the absence of other metabolic bone disorders, and even with a normal bone mineral density (T-score). OP is also diagnosed based on a T-score of − 2.5 or lower, even in the absence of a prevalent fracture^10^. Patients were excluded if they exhibited: 1) Age < 50 years (n = 73). 2). Patients who fasted for less than 8 h to undergo fasting blood glucose (FBG) testing (n = 54). 3). Patients suffering from medical conditions or taking medications that interfere with glucose metabolism (n = 125). 4). Patients with incomplete data (n = 73). A total of 2084 patients did not meet exclusion criteria and were enrolled in the study. In this study, we did not focus on whether or not patients had been diagnosed with diabetes, and as such the diagnosis of diabetes had no impact on this study of the association between 25(OH)D and blood glucose levels. A schematic diagram of our patient selection process is presented in Fig. 1. We received ethical approval from the Affiliated Kunshan Hospital of Jiangsu University (approval No. 2020-03-046-K01), and the study was compliant with the Declaration of Helsinki. Patient information was initially documented for the hospital’s quality improvement purposes. Data analyzers were blinded to the identity of patients. Written informed consent was not required due to the anonymous data gathering and observational nature of the study.

### Dependent variable

FBG is the dependent variable of this study. It has important significance for diabetes and can be conveniently and objectively observed. FBG were measured on an empty stomach (at least 8 h) to ensure the accuracy of blood glucose. In clinical settings, the FBG is strongly recommended because it is easier and faster to perform, more convenient and acceptable to patients, more reproducible, and less expensive.

### Measurement of vitamin D

In humans, the most abundant form of vitamin D in the blood is 25(OH)D, and serum levels are used to reliably estimate a patient’s vitamin D status. Serum 25(OH)D concentrations were measured immediately using an automated electrochemiluminescence immunoassay on a Roche Cobas 8000/e602 analyzer (Roche Diagnostics, Mannheim, Germany). There is no medical consensus regarding the status cut-off values of 25(OH)D concentrations. Vitamin D deficiency is defined as a 25(OH)D below 20 ng/ml (50 nmol/L), and vitamin D insufficiency as a 25(OH)D of 21–29 ng/ml (52.5–72.5 nmol/L)^11^. The time of blood collection was included in the analysis. Seasons were defined as: Spring, March–May; Summer, June–August; Autumn, September–November; Winter, December–February.

### Laboratory tests

All of the laboratory tests were analyzed using the Wako Assay Kit loaded on the LABOSPECT 008 automated analyzer (Hitachi High-Technologies, Tokyo, Japan). FBG were measured using the hexokinase glucose-6-phosphate dehydrogenase method and measured by an automatic biochemical analyzer (Accu-Chek Guide; Roche Diabetes Care GmbH, Germany).

### Covariates

The covariates analyzed in this study were age, sex, body mass index (BMI), season of blood collection, year of blood collection, neutrophil count (reference range: 2–7.5 × 10^9^/L), lymphocyte count (reference range: 2–4 × 10^9^/L), category of diagnosis (OP without OPF, OPF), hemoglobin (reference range: male: 120–165 g/L, female: 110–150 g/L), calcium level (reference range: 2.25–2.75 mmol/L), albumin level (reference range: 35–50 g/L), high-density lipoprotein (HDL) level (reference range: 1.16–1.55 mmol/L), and the Charlson comorbidity index (CCI) score 12. All laboratory variables were measured after hospitalization, and patients were asked to fast before the collection of blood samples. Comorbidity was assessed using the CCI score.

### Statistics

Patient demographic, clinical, and laboratory characteristics are expressed as mean (standard deviation [SD]) or/and median (first quartile [Q1] to third quartile [Q3]) in case of continuous variables, and as frequency (percentage) in the case of categorical variables; *P*-value on ANOVA, *P*-value* on Kruskal–Wallis Rank Test for continuous variables or Fisher Exact for categorical variables with Expected < 10. A univariate analysis was carried out with the Pearson’s chi-square or Fisher’s exact test in case of categorical data. Independent samples t-test was employed for continuous variables with normal distribution, and Mann–Whitney U test was employed for non-normally distributed continuous data. We also conducted univariate logistic regression analysis to evaluate the association between the characteristics of OP patients and FBG.

Using generalized estimating equations (GEE) allowed us to evaluate independent associations between FBG and maternal serum 25(OH)D by controlling for the influence of covariances. We calculated the results of the unadjusted or minimally adjusted analysis and those from fully adjusted analysis. First, collinearity diagnosis of covariances was performed using variance inflation factor (VIF) analysis (the variable average total cholesterol was first removed due to VIF > 10). Then, a judgement on whether to adjust covariances was made using the following principles: Criteria 1, the covariate is added to the basic model or removed from the full model and the matched odds ratio (OR) or standard regression coefficient (β) is changed by at least 10%; Criteria 2: Criteria 1 or a covariate *P*-value of < 0.1 in the univariate model.

Non-linear relationships were additionally identified via a generalized additive model (GAM), and on finding a non-linear correlation, the threshold effect in terms of the smoothing curve was calculated using a two-piecewise linear regression model. When a clear ratio was apparent in the smoothing curve, the recursive method was applied to automatically calculate the turning point at which to use the maximum likelihood model.

In addition, to test the robustness and potential variation in the different subgroups, we repeated the subgroup analyses while stratifying by different covariates.

All statistical analyses were performed using the Empower Stats (www.empowerstats.com, X&Y solutions, Inc., Boston, MA, USA) and R software version 3.6.3 (http://www.r-project.org). A *P*-value < 0.05 was set as the significance threshold.

## Results

### Patients characteristics

There were 2,084 patients included in final analysis. 1089 patients had serum 25(OH)D of 20 ng/ml or less, 718 patients had serum 25(OH)D between 20 and 30 ng/ml, while the others had serum 25(OH)D above 30 ng/ml. Patients with vitamin D deficiency had the highest mean FBG, at 6.29 mmol/L. Patients with serum 25(OH)D above 30 ng/ml had the lowest fasting glucose (Mean, 5.77; SD, 1.58; mmol/L). Women are more likely than men to be deficient in vitamin D. With the exception of the lymphocyte count and the year of blood drawing, it appeared that all variables were statistically significant with respect to serum 25(OH)D levels (Table 1).

**Table 1:** Characteristics of study participants.

Variables^a^	25(OH)D < = 20 ng/mL	25(OH)D > 20, < = 30 ng/mL	25(OH)D > 30 ng/mL	*P*-value	*P*-value*
	(N) Mean (SD) or Median (Q1–Q3)/N(%)	(N) Mean (SD) or Median (Q1–Q3) / N(%)	(N) Mean (SD) or Median (Q1–Q3) / N(%)		
N	1089	718	277		
FBG, mmol/L	(1089) 6.29 (2.08)	(718) 5.89 (1.86)	(277) 5.77 (1.58)	< 0.001	< 0.001
Age, y	(1089) 69.28 (8.75)	(718) 67.46 (8.24)	(277) 68.33 (9.03)	< 0.001	< 0.001
BMI, kg/m^2^	(1089) 23.65 (3.28)	(718) 23.33 (2.97)	(277) 22.88 (2.76)	< 0.001	0.001
Hemoglobin, g/L	(1046) 122.20 (17.02)	(698) 125.15 (15.04)	(271) 126.62 (16.16)	< 0.001	< 0.001
Albumin, g/L	(1072) 39.85 (4.75)	(704) 40.68 (4.56)	(269) 41.04 (4.86)	< 0.001	< 0.001
Calcium, mmol/L	(1072) 2.25 (0.18)	(705) 2.27 (0.16)	(272) 2.28 (0.15)	< 0.001	< 0.001
Neutrophil count, × 10^9^/L	(1046) 4.52 (2.36)	(697) 4.17 (2.25)	(271) 3.94 (2.53)	< 0.001	< 0.001
Lymphocyte count, × 10^9^/L	(1046) 1.41 (0.56)	(697) 1.43 (0.54)	(271) 1.50 (0.58)	0.074	0.047
High Density Lipoprotein, mmol/L	(811) 1.44 (0.32)	(556) 1.49 (0.35)	(219) 1.52 (0.33)	< 0.001	< 0.001
Sex, N (%)				< 0.001	–
Male	136 (12.49%)	122 (16.99%)	77 (27.80%)		
Female	953 (87.51%)	596 (83.01%)	200 (72.20%)		
Category of diagnosis				< 0.001	–
OP without OPF	646 (59.32%)	542 (75.49%)	224 (80.87%)		
OPF	443 (40.68%)	176 (24.51%)	53 (19.13%)		
Supplementing Vitamin D categorical				0.613	–
Supplementing Vitamin D	114 (10.47%)	66 (9.19%)	30 (10.83%)		
Not Supplementing Vitamin D	975 (89.53%)	652 (90.81%)	247 (89.17%)		
Season of blood collection, N (%)				< 0.001	–
Spring (March, April and May)	320 (29.38%)	141 (19.64%)	56 (20.22%)		
Summer (June, July and August)	251 (23.05%)	189 (26.32%)	76 (27.44%)		
Autumn (September, October and November)	264 (24.24%)	242 (33.70%)	102 (36.82%)		
Winter (December, January and February)	254 (23.32%)	146 (20.33%)	43 (15.52%)		
Year of blood collection, N (%)				0.051	–
2015	15 (1.38%)	3 (0.42%)	3 (1.08%)		
2016	15 (1.38%)	13 (1.81%)	4 (1.44%)		
2017	18 (1.65%)	23 (3.20%)	7 (2.53%)		
2018	72 (6.61%)	41 (5.71%)	15 (5.42%)		
2019	260 (23.88%)	158 (22.01%)	61 (22.02%)		
2020	269 (24.70%)	227 (31.62%)	82 (29.60%)		
2021	388 (35.63%)	228 (31.75%)	89 (32.13%)		
2022	52 (4.78%)	25 (3.48%)	16 (5.78%)		
CCI				0.017	–
0	782 (71.81%)	482 (67.13%)	171 (61.73%)		
1	121 (11.11%)	106 (14.76%)	39 (14.08%)		
2	40 (3.67%)	34 (4.74%)	12 (4.33%)		
3	43 (3.95%)	47 (6.55%)	22 (7.94%)		
4	39 (3.58%)	14 (1.95%)	17 (6.14%)		
5	5 (0.46%)	3 (0.42%)	1 (0.36%)		
6	9 (0.83%)	6 (0.84%)	5 (1.81%)		
7	5 (0.46%)	1 (0.14%)	0 (0.00%)		
8	19 (1.74%)	9 (1.25%)	5 (1.81%)		
9	4 (0.37%)	6 (0.84%)	0 (0.00%)		
10	6 (0.55%)	1 (0.14%)	2 (0.72%)		
11	5 (0.46%)	5 (0.70%)	1 (0.36%)		
12	5 (0.46%)	2 (0.28%)	0 (0.00%)		
13	5 (0.46%)	1 (0.14%)	2 (0.72%)		
14	1 (0.09%)	1 (0.14%)	0 (0.00%)		

Result: (N) Mean(SD) Median (Q1–Q3) / N(%).

^a^FBG, hemoglobin, albumin, calcium and high density lipoprotein referred to the concentrations in the serum. Neutrophil count and lymphocyte count referred to count in peripheral blood.

*P*-value: ANOVA.

*P*-value*: Kruskal Wallis Rank Test for continuous variables, Fisher Exact for categorical variables with Expects < 10.

FBG, fasting blood glucose; 25(OH)D, 25-hydroxy vitamin D; BMI, body mass index; CCI, Charlson comorbidity index; OP, osteoporosis; OPF, osteoporosis in fracture patients.

### Univariate analysis

Furthermore, we performed univariate analysis, as shown in Table 2. We found that serum 25(OH)D levels were inversely associated with FBG below 20 ng/ml, whereas above 20 ng/ml, FBG values did not appear to have a statistically significant relationship with serum 25(OH)D without adjusting for variables. We also found that hemoglobin content, albumin, serum calcium, lymphocytes, neutrophils, and high-density lipoprotein had significant significance for the change of FBG in univariate analysis.

**Table 2:** Univariate analysis for FBG.

Variables^a^	25(OH)D < = 20 ng/mL	25(OH)D > 20, < = 30 ng/mL	25(OH)D > 30 ng/mL	Total
	OR/β (95% CI) *P*-value	OR/β (95% CI) *P*-value	OR/β (95% CI) *P*-value	OR/β (95% CI) *P*-value
25(OH)D, ng/mL	− 0.05 (− 0.08, − 0.01) 0.0065	− 0.03 (− 0.08, 0.02) 0.2449	0.01 (− 0.02, 0.03) 0.4748	− 0.02 (− 0.04, 0.00) 0.0607
Age, y	0.00 (− 0.01, 0.02) 0.6149	0.03 (0.01, 0.04) 0.0023	0.01 (− 0.01, 0.03) 0.3395	0.01 (0.00, 0.02) 0.0201
BMI, kg/m^2^	0.05 (0.02, 0.09) 0.0045	0.04 (− 0.01, 0.08) 0.1230	0.07 (0.01, 0.14) 0.0342	0.05 (0.02, 0.08) 0.0002
Sex, N (%)				
Male	Reference	Reference	Reference	Reference
Female	0.20 (− 0.18, 0.57) 0.3002	− 0.69 (− 1.05, − 0.33) 0.0002	− 0.03 (− 0.45, 0.39) 0.8917	− 0.18 (− 0.40, 0.05) 0.1357
Hemoglobin, g/L	− 0.01 (− 0.02, − 0.00) 0.0031	− 0.00 (− 0.01, 0.01) 0.8332	− 0.00 (− 0.02, 0.01) 0.4096	− 0.01 (− 0.01, − 0.00) 0.0063
Albumin, g/L	− 0.04 (− 0.06, − 0.01) 0.0044	− 0.04 (− 0.07, − 0.01) 0.0062	0.01 (− 0.03, 0.04) 0.7645	− 0.03 (− 0.05, − 0.02) 0.0003
Calcium, mmol/L	− 0.79 (− 1.49, − 0.10) 0.0259	− 0.96 (− 1.83, − 0.09) 0.0318	− 0.76 (− 1.93, 0.41) 0.2059	− 0.84 (− 1.34, − 0.34) 0.0010
Neutrophil count, × 10^9^/L	0.20 (0.15, 0.25) < 0.0001	0.18 (0.12, 0.24) < 0.0001	0.09 (0.01, 0.16) 0.0193	0.18 (0.14, 0.21) < 0.0001
Lymphocyte count, × 10^9^/L	− 0.03 (− 0.25, 0.19) 0.7940	− 0.31 (− 0.56, − 0.06) 0.0156	− 0.26 (− 0.58, 0.06) 0.1149	− 0.15 (− 0.30, − 0.01) 0.0424
High Density Lipoprotein, mmol/L	− 0.46 (− 0.87, − 0.05) 0.0277	− 0.54 (− 0.91, − 0.16) 0.0052	− 0.27 (− 0.76, 0.22) 0.2766	− 0.46 (− 0.72, − 0.21) 0.0003
Category of diagnosis				
OP without OPF	Reference	Reference	Reference	Reference
OPF	0.60 (0.35, 0.85) < 0.0001	1.30 (1.00, 1.61) < 0.0001	0.81 (0.35, 1.28) 0.0007	0.83 (0.65, 1.01) < 0.0001
Season of blood collection, N (%)				
Spring (March, April and May)	Reference	Reference	Reference	Reference
Summer (June, July and August)	0.02 (− 0.33, 0.36) 0.9294	0.38 (− 0.02, 0.79) 0.0641	− 0.18 (− 0.73, 0.37) 0.5150	0.10 (− 0.14, 0.34) 0.4099
Autumn (September, October and November)	− 0.02 (− 0.36, 0.32) 0.8905	0.33 (− 0.05, 0.72) 0.0908	− 0.13 (− 0.65, 0.38) 0.6115	0.07 (− 0.16, 0.30) 0.5336
Winter (December, January and February)	0.19 (− 0.16, 0.53) 0.2875	0.62 (0.19, 1.05) 0.0049	− 0.28 (− 0.91, 0.35) 0.3893	0.27 (0.02, 0.51) 0.0358
Year of blood collection, N (%)				
2015	Reference	Reference	Reference	Reference
2016	1.67 (0.20, 3.15) 0.0265	0.76 (− 1.58, 3.11) 0.5250	0.28 (− 2.09, 2.66) 0.8154	0.95 (− 0.12, 2.01) 0.0831
2017	− 0.46 (− 1.88, 0.95) 0.5236	0.48 (− 1.77, 2.72) 0.6786	− 0.37 (− 2.52, 1.77) 0.7341	− 0.23 (− 1.22, 0.77) 0.6573
2018	− 0.48 (− 1.63, 0.67) 0.4140	0.59 (− 1.60, 2.78) 0.5990	− 0.84 (− 2.81, 1.13) 0.4038	− 0.31 (− 1.20, 0.59) 0.5004
2019	0.23 (− 0.85, 1.30) 0.6808	0.89 (− 1.25, 3.02) 0.4154	− 0.02 (− 1.86, 1.82) 0.9814	0.28 (− 0.57, 1.13) 0.5168
2020	− 0.12 (− 1.20, 0.95) 0.8230	0.67 (− 1.46, 2.79) 0.5385	− 0.20 (− 2.03, 1.63) 0.8299	0.01 (− 0.84, 0.85) 0.9861
2021	0.26 (− 0.80, 1.33) 0.6267	0.90 (− 1.23, 3.02) 0.4089	− 0.17 (− 2.00, 1.66) 0.8544	0.29 (− 0.56, 1.13) 0.5055
2022	0.64 (− 0.55, 1.83) 0.2901	1.04 (− 1.20, 3.28) 0.3618	− 0.66 (− 2.62, 1.29) 0.5066	0.45 (− 0.47, 1.37) 0.3391
CCI				
0	Reference	Reference	Reference	Reference
1	0.30 (− 0.09, 0.70) 0.1352	− 0.36 (− 0.75, 0.04) 0.0769	0.31 (− 0.24, 0.86) 0.2731	0.05 (− 0.20, 0.31) 0.6930
2	− 0.19 (− 0.84, 0.47) 0.5794	− 0.21 (− 0.86, 0.44) 0.5240	− 0.35 (− 1.28, 0.58) 0.4600	− 0.21 (− 0.63, 0.21) 0.3337
3	− 0.02 (− 0.66, 0.61) 0.9422	− 0.44 (− 1.00, 0.12) 0.1207	0.27 (− 0.43, 0.97) 0.4483	− 0.13 (− 0.50, 0.25) 0.5061
4	0.39 (− 0.27, 1.06) 0.2438	0.27 (− 0.72, 1.26) 0.5947	0.16 (− 0.63, 0.95) 0.6890	0.29 (− 0.17, 0.76) 0.2202
5	2.80 (0.98, 4.61) 0.0026	− 0.40 (− 2.52, 1.72) 0.7133	0.04 (− 3.07, 3.15) 0.9802	1.42 (0.15, 2.70) 0.0285
6	0.60 (− 0.76, 1.95) 0.3873	− 0.02 (− 1.53, 1.48) 0.9764	0.57 (− 0.84, 1.97) 0.4301	0.40 (− 0.46, 1.26) 0.3609
7	1.57 (− 0.24, 3.38) 0.0903	− 0.68 (− 4.35, 2.99) 0.7159	NA	1.17 (− 0.39, 2.72) 0.1430
8	− 0.55 (− 1.49, 0.39) 0.2519	− 0.96 (− 2.19, 0.28) 0.1285	− 0.02 (− 1.43, 1.39) 0.9772	− 0.59 (− 1.26, 0.08) 0.0848
9	− 0.35 (− 2.38, 1.68) 0.7364	− 0.23 (− 1.73, 1.28) 0.7668	NA	− 0.23 (− 1.44, 0.97) 0.7040
10	0.11 (− 1.54, 1.77) 0.8924	1.33 (− 2.34, 5.00) 0.4777	− 1.97 (− 4.17, 0.24) 0.0819	− 0.25 (− 1.52, 1.03) 0.7026
11	− 0.49 (− 2.31, 1.32) 0.5932	0.11 (− 1.54, 1.75) 0.8987	2.58 (− 0.53, 5.69) 0.1053	0.08 (− 1.08, 1.23) 0.8956
12	2.58 (0.77, 4.40) 0.0053	1.12 (− 1.48, 3.71) 0.3983	NA	2.16 (0.71, 3.60) 0.0035
13	2.38 (0.57, 4.20) 0.0102	− 0.95 (− 4.62, 2.72) 0.6113	− 0.99 (− 3.20, 1.22) 0.3797	1.09 (− 0.26, 2.44) 0.1138
14	3.24 (− 0.81, 7.29) 0.1169	− 1.95 (− 5.62, 1.72) 0.2973	NA	0.67 (− 2.03, 3.36) 0.6264

^a^FBG, hemoglobin, albumin, calcium and high-density lipoprotein referred to the concentrations in the serum. Neutrophil count and lymphocyte count referred to count in peripheral blood.

FBG, fasting blood glucose; OR, odds ratio; β, standard regression coefficient; CI, confidence interval; 25(OH)D, 25-hydroxy vitamin D; BMI, body mass index; CCI, Charlson comorbidity index; OP, osteoporosis in non-fracture patients; OPF, osteoporosis in fracture patients; NA, not applicable.

### Multivariate linear regression analysis

Table 3 show the independent association between serum 25(OH)D and FBG using multivariate linear regression analysis. For all patients’ serum 25(OH)D was significantly negatively associated with FBG in the unadjusted model (β, − 0.02; 95% CI, − 0.03, − 0.02; *P* < 0.0001). And after adjusting age, sex, BMI, neutrophil count, category of diagnosis, season of blood collection and year of blood collection, the negative association was significant (β, − 0.01; 95% CI − 0.02, − 0.00; *P* = 0.0473). Further adjusting hemoglobin, calcium, albumin, high density lipoprotein, lymphocyte count and CCI, the negative association was significant (β, − 0.02; 95% CI, − 0.03, − 0.01; *P* = 0.0011).

**Table 3:** Relationship between serum 25(OH)D level and FBG in different models.

	Crude Model^a^		Model I^b^		Model II^c^	
	β (95% CI)	*P*-value	β (95% CI)	*P*-value	β (95% CI)	*P*-value
25(OH)D, ng/mL	− 0.02 (− 0.03, − 0.02)	< 0.0001	− 0.01 (− 0.02, − 0.00)	0.0473	− 0.02 (− 0.03, − 0.01)	0.0011

^a^No adjustment.

^b^Adjusted for sex, age, BMI, neutrophil count, category of diagnosis, season of blood collection and year of blood collection.

^c^Adjusted for Model I plus hemoglobin, calcium, albumin, high density lipoprotein, lymphocyte count and CCI.

FBG, fasting blood glucose; β, standard regression coefficient; CI, confidence interval; 25(OH)D, 25-hydroxy vitamin D; BMI, body mass index; CCI, Charlson comorbidity index.

### Threshold analysis and the spline smoothing plot

**Figure 2:**
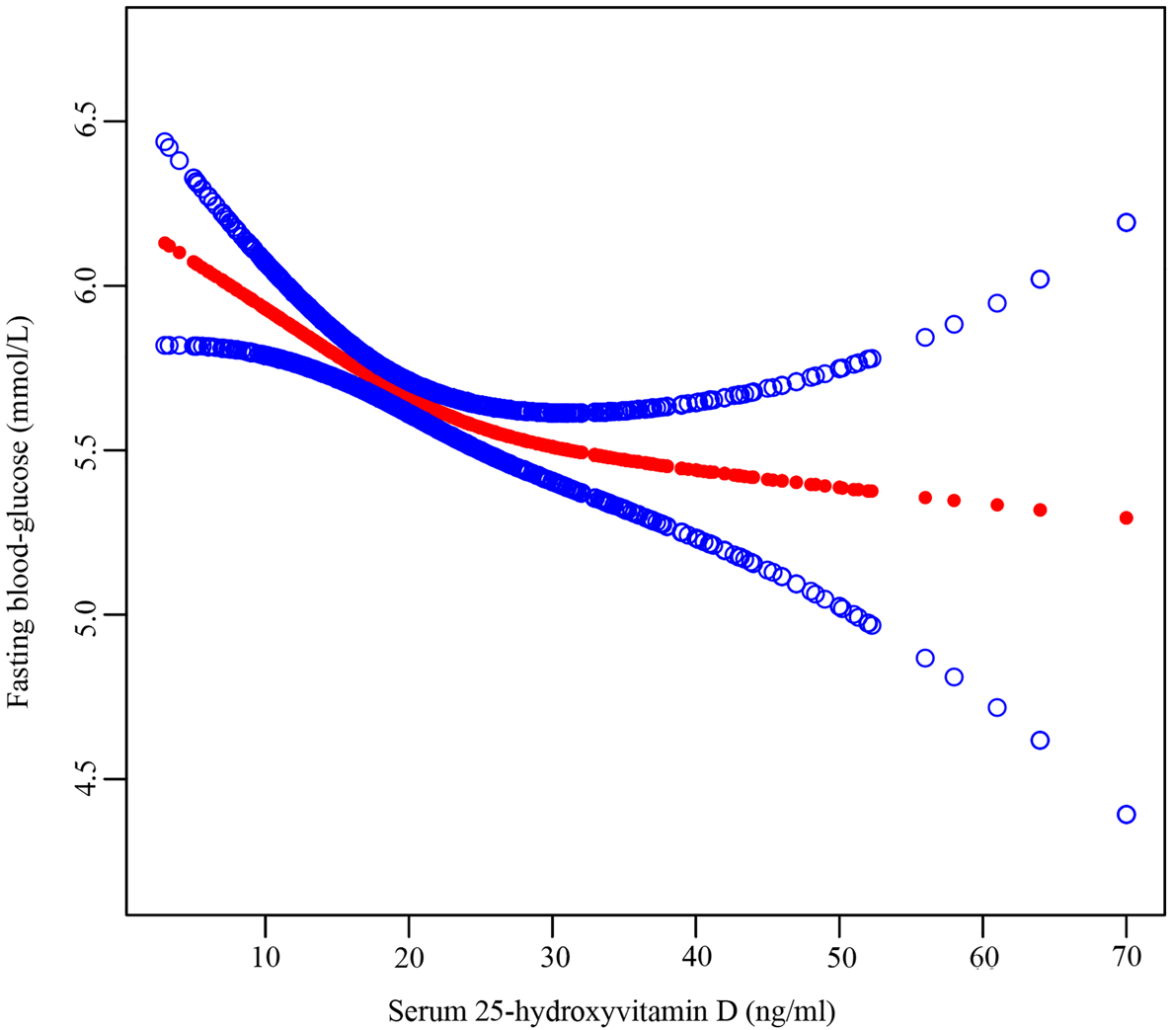
The adjusted smoothed curves of the 25(OH)D and FBG. In this plot, the red line indicates the nonlinear association between 25(OH)D and FBG, and the blud lines serve as 95% confidence interval A nonlinear relationship was observed after adjusting for age, gender, BMI, neutrophil count, diagnosis categorical, season of blood collection, year of blood collection, hemoglobin, calcium, albumin, high density lipoprotein, lymphocyte count and CCI.

The data in Table 4 correspond to threshold effect analyses assessing the association between serum 25(OH)D and FBG levels. These analyses were adjusted for age, sex, BMI, neutrophil count, category of diagnosis, season of blood collection, year of blood collection, hemoglobin, calcium, albumin, high density lipoprotein, lymphocyte count and CCI, and ultimately revealed a significant non-linear relationship with an inflection point (K) at 23.39 ng/mL. This indicates that at serum 25(OH)D level below this threshold, a strong significant negative relationship is evident between serum 25(OH)D level and glucose (β, − 0.04; 95% CI, − 0.05, − 0.02; *P* = 0.0002). In contrast, when serum 25(OH)D levels exceed 23.39 ng/mL, these two variables are not significantly related (β, − 0.00; 95% CI − 0.02, 0.02; *P* = 0.9616) (Fig. 2).

**Table 4:** Threshold effect analysis examining the relationship between serum 25(OH)D level and FBG.

	Model II^a^	*P*-value
	β (95% CI)	
Model A^b^		
One line slope	− 0.02 (− 0.03, − 0.01)	0.0011
Model B^c^		
Serum 25(OH)D turning point (K), ng/mL	23.39	
< K	− 0.04(− 0.05, − 0.02)	0.0002
> K	− 0.00 (− 0.02, 0.02)	0.9616
Slope 2 – Slope 1	0.03(0.01, 0.06)	0.0185
LRT^d^		0.0170

^a^Adjusted for sex, age, BMI, neutrophil count, category of diagnosis, season of blood collection, year of blood collection, hemoglobin, calcium, albumin, high density lipoprotein, lymphocyte count and CCI.

^b^Linear analysis, *P*-value < 0.05 indicates a linear relationship.

^c^Non-linear analysis.

^d^*P*-value < 0.05 means Model B is significantly different from Model A, which indicates a non-linear relationship.

Abbreviations: FBG, fasting blood glucose; β, standard regression coefficient; CI, confidence interval; 25(OH)D, 25-hydroxy vitamin D; K, threshold; LRT, likelihood ratio test, BMI, body mass index; CCI, Charlson comorbidity index.

### Subgroup analysis

To further confirm that the findings are robust to potential confounders in fully adjusted Model II, we performed the subgroup analyses while stratifying by sex, age, BMI, neutrophil count, hemoglobin, albumin, lymphocyte count, high density lipoprotien, category of diagnosis, supplementing vitamin D categorical, season of blood collection, year of blood collection and ICC (Table 5). All analysis were adjusted for the above ten covariates except the subgroup variable. We found significant interactions (Table S1) between the associations of hemoglobin with blood glucose and vitamin D in both unadjusted and incomplete adjusted models (*P* = 0.0280; *P* = 0.0175), with changes in vitamin D and blood glucose being more pronounced when hemoglobin was less than 110 g/L (β, − 0.05; 95% CI − 0.07–0.02; *P* = 0.0002; β, − 0.04; 95% CI − 0.06–0.01; *P* = 0.0037). However, in the fully adjusted model, the interaction was not significant (*P* = 0.0972). We did not find any significant interaction effects between exposed factors (i.e. gender, Season of blood collection, year of blood collection, Neutrophil count, Main diagnosis, BMI, Hemoglobin, Calcium, Albumin, High Density Lipoprotein, CCI and Lymphocyte count. all *P* ≥ 0.05).

**Table 5:** Subgroup analyses examining the relationship between serum 25(OH)D level and FBG.

Subgroup	N	OR (95% CI)	*P*-value	*P*-value for interaction
Sex				0.1248
Male	335	− 0.01 (− 0.03, 0.02)	0.5013	
Female	1749	− 0.03 (− 0.04, − 0.02)	< 0.0001	
Age tertile				0.3803
Tertile 1 (50–64 y)	673	− 0.03 (− 0.05, − 0.01)	0.0002	
Tertile 2 (65–71 y)	680	− 0.03 (− 0.05, − 0.01)	0.0004	
Tertile 3 (72–94 y)	731	− 0.01 (− 0.03, 0.00)	0.1021	
BMI categorical kg/m^2^				0.5220
< 18.5	100	− 0.04 (− 0.07, 0.00)	0.0832	
> = 18.5, < 24	1272	− 0.02 (− 0.03, − 0.01)	0.0011	
> = 24	712	− 0.03 (− 0.05, − 0.01)	0.0004	
Neutrophil count, × 10^9^/L				0.3052
< = 1.8	97	0.00 (− 0.03, 0.04)	0.8774	
> 1.8, < = 6.8	1638	− 0.02 (− 0.03, − 0.01)	< 0.0001	
> 6.8	279	− 0.02 (− 0.05, 0.02)	0.3715	
Hemoglobin, g/L				0.0280
< 110	284	− 0.05 (− 0.08, − 0.02)	0.0014	
> = 110	1731	− 0.02 (− 0.03, − 0.01)	0.0003	
Albumin, g/L				0.3604
< 28	29	− 0.07 (− 0.16, 0.01)	0.1064	
> = 28, < 44	1551	− 0.03 (− 0.04, − 0.01)	< 0.0001	
> = 44	465	− 0.03 (− 0.05, − 0.02)	< 0.0001	
Lymphocyte count, × 10^9^/L				0.9618
< 0.8	220	− 0.02 (− 0.06, 0.01)	< 0.0001	
> = 0.8, < 4	1792	− 0.02 (− 0.03, − 0.01)	< 0.0001	
> = 4	2			
High Density Lipoprotien, mmol/L				0.3341
< 1.16	262	− 0.02 (− 0.05, 0.01)	0.2905	
> = 1.16, < 1.55	737	− 0.03 (− 0.04, − 0.01)	0.0001	
> = 1.55	587	− 0.01 (− 0.03, − 0.00)	0.0223	
Category of diagnosis				0.0566
OP without OPF	1412	− 0.02 (− 0.03, − 0.01)	< 0.0001	
OPF	672	0.00 (− 0.02, 0.03)	0.7202	
Supplementing Vitamin D categorical				0.0764
Supplementing Vitamin D	203	− 0.03 (− 0.06, − 0.01)	0.0083	
Not Supplementing Vitamin D	1789	− 0.01 (− 0.02, 0.00)	0.0666	
Season of blood collection				0.8578
Spring (March, April and May)	517	− 0.02 (− 0.04, − 0.00)	0.0267	
Summer (June, July and August)	516	− 0.03 (− 0.05, − 0.01)	0.0045	
Autumn (September, October and November)	608	− 0.02 (− 0.04, − 0.00)	0.0323	
Winter (December, January and February)	443	− 0.03 (− 0.05, − 0.01)	0.0323	
Year				0.3832
2015	21	− 0.01 (− 0.07, 0.04)	0.5816	
2016	32	− 0.05 (− 0.17, 0.08)	0.4832	
2017	48	− 0.01 (− 0.08, 0.06)	0.4832	
2018	128	− 0.02 (− 0.05, 0.02)	0.3496	
2019	479	− 0.02 (− 0.05, − 0.00)	0.0290	
2020	578	− 0.01 (− 0.03, 0.00)	0.0862	
2021	705	− 0.02 (− 0.04, − 0.01)	0.0043	
2022	93	− 0.07 (− 0.11, − 0.03)	0.0010	
ICC				0.9274
0	1435	− 0.02 (− 0.04, − 0.01)	< 0.0001	
1	266	− 0.03 (− 0.06, 0.01)	0.1235	
2	86	− 0.03 (− 0.07, 0.01)	0.1842	
3	112	− 0.01 (− 0.04, 0.02)	0.5573	
4	70	− 0.01 (− 0.06, 0.03)	0.5300	
5	9			
6	20	− 0.01 (− 0.07, 0.05)	0.6803	
7	6			
8	33	0.01 (− 0.05, 0.08)	0.6732	
9	10			
10	9			
11	11	0.06 (− 0.10, 0.22)	0.4904	
12	7			
13	8			
14	2			

^a^Adjusted for sex, age, BMI, neutrophil count, hemoglobin, albumin, lymphocyte count, high density lipoprotien, category of diagnosis, supplementing vitamin D categorical, season of blood collection, year of blood collection and ICC except the subgroup variable.

Abbreviations: OR, odds ratio; β, standard regression coefficient; CI, confidence interval; 25(OH)D, 25-hydroxy vitamin D; FBG, fasting blood glucose; BMI, body mass index; CCI, Charlson comorbidity index; OP, osteoporosis; OPF, osteoporosis in fracture patients.

## Discussion

In this retrospective cross-section study, we found that in the osteoporotic population, we explored the correlation between serum 25(OH)D and FBG, and found that when adjusting for any factor, FBG concentration decreased by 0.02 mmol/L for every 1 ng/ mL increase in serum 25(OH)D concentration. In the adjusted 2 model, FBG decreased by 0.02 mmol/L for every 1 ng/mL increase in serum 25(OH)D concentration. Subsequently, we performed curve fitting and threshold effect value analysis, and found that fasting glucose and serum 25(OH)D were not only linear, but also non-linear. When the concentration of 25(OH)D was less than 23.39 ng/mL, the relationship between the two was most significant, and the 25(OH)D increased by 1 ng/mL, and the FBG decreased by 0.04 mmol/L. However, when the concentration of 25(OH)D was greater than 23.39 ng/ mL, the relationship between the two was not obvious. Therefore, we suggest that vitamin D supplementation in patients with OP should reach 23.39 ng/ mL, which may reduce blood glucose. In the end, we complete subgroup analyses examining the relationship between serum 25(OH)D level and glucose. The relationship between FBG and 25(OH)D was stable. But it also found that hemoglobin may be mediated by interaction (Table S1).

The few studies that have investigated the relationship between FBG levels and 25(OH)D have found no relationship. Intraperitoneal injections of vitamin D (7 ng/g) administered daily for 15 days to female albino mice with alloxan-induced diabetes led to significant improvements in glucose metabolism. Specifically, the treatment resulted in lower serum glucose levels, enhanced activity of enzymes involved in glucose metabolic pathways, restoration of glucose homeostasis, and reduced pancreatic and liver damage, according to animal studies^12^. Administration of 20,000 IU/kg cholecalciferol via intraperitoneal injection in male Sprague–Dawley rats with streptozotocin-induced diabetes led to significant improvements in metabolic markers. Specifically, the treatment resulted in decreased levels of fasting plasma glucose (FBG) and Hemoglobin A1c (HbA1c), as well as improved levels of insulin and IGF-1^13^. In human studies, in the study of Mohammad J. Alkhatatbeh and Khalid K. Abdul-Razzak^14^, a significant negative correlation was found between HbA1c and 25(OH)D levels, no similar association was found between FBG and 25(OH)D levels in patients with T2DM. In their cohort study, Zoppini et al. found no significant correlation between 25-hydroxyvitamin D (25(OH)D) levels and fasting blood glucose (FBG) levels^15^. Wu Chunhua et al.^16^ found that vitamin D supplementation had no effect on FBG levels in patients with T2DM. In our research, we found that FBG decreased with the increase of serum 25(OH)D. This may be because of the data we have in the OP population. Endocrine society clinical guidelines recommend maintaining serum 25 hydroxyvitamin D at approximately 30 ng/ml^11^. In a double-blind randomized clinical trial, weekly treatment with 50,000 IU oral vitamin D for eight weeks in vitamin D-deficient obese individuals with type 2 diabetes mellitus (T2DM) led to a significant decrease in HbA1c levels, but did not result in significant changes in fasting blood glucose (FBG), insulin, HOMA-IR index, or quantitative insulin sensitivity check index (QUICKI)^17^. However, we recommend that maintaining serum 25 hydroxyl vitamin D at 23 ng/ mL may be the most effective for lowering blood glucose, while it has no effect when it exceeds 23 ng/ml.

The involvement of vitamin D in glucose metabolism is thought to be linked to its role in pancreatic insulin secretion and regulation of peripheral insulin sensitivity^18^. Although commonly known as a vitamin, vitamin D has been recognized to have several hormonal functions that are thought to occur through its interactions with vitamin D receptors (VDRs). These receptors are widely expressed on various cell types and play a key role in mediating the effects of vitamin D on target tissues^19^. One of these functions is the interaction of vitamin D with VDRs present in pancreatic beta islet cells^19^. Studies in animal models have shown that mice lacking functional vitamin D receptors (VDRs) exhibit impaired insulin secretion^20^. Moreover, studies have shown that vitamin D supplementation can stimulate insulin biosynthesis in pancreatic islets of rats^21^. In humans, certain variants of the VDR gene have been linked to impaired insulin secretion and an increased risk of developing type 2 diabetes mellitus (T2DM)^22^. Furthermore, studies suggest that vitamin D may play a role in enhancing peripheral insulin sensitivity, possibly through its interactions with VDRs expressed in human skeletal muscle and adipose tissue cells^18,23,24^. Specifically, skeletal muscle and adipose tissue cells play a crucial role in determining peripheral insulin sensitivity, as they are responsible for glucose uptake in response to insulin secretion^18^. Vitamin D both enhances and promotes insulin secretion from pancreatic β-cells insulin. One of the underlying molecular mechanisms involves the regulation of intracellular Ca^2+^ concentrations. Non-genomic actions of 1, 25-hydroxyvitamin D3 (1,25[OH]2D3) have been identified as responsible for increasing cytoplasmic Ca^2+^ levels, leading to activation of insulin exocytosis in pancreatic β-cells and subsequent increased insulin secretion^25,26^. Vitamin D deficiency can contribute to insulin resistance through several potential mechanisms. For example, the activation of peroxisome proliferator-activated receptor delta (PPAR-δ) by 1,25(OH)2D3 enhances insulin sensitivity^27^. Additionally, vitamin D improves glucose metabolism by upregulating the Sirtuin 1 (SIRT1)/ insulin receptor substrate1 (IRS1)/ Glucose transporter type 4 (GLUT-4)signaling cascade and enhancing glucose uptake, which is especially evident in high glucose-treated C2C12 mouse myoblast cell (C2C12) myotubes^28^. Vitamin D also modulates the low-grade chronic inflammation often associated with insulin resistance. Notably, pro-inflammatory cytokines such as Tumor Necrosis Factor-alpha (TNF-α) interfere with peripheral insulin sensitivity by inhibiting insulin-dependent tyrosine phosphorylation of IRS-1. This disrupts the appropriate activation of downstream insulin signaling molecules, including Phosphatidylinositol 3-kinase (PI3K), and the translocation of GLUT-4 to the cell surface^29,30^. Vitamin D counteracts the release of pro-inflammatory cytokines, such as TNF-α and interleukin 6 (IL-6), and C-reactive protein^31^. Furthermore, the bioactive form of vitamin D strongly suppresses the activation of the nuclear factor kappa-B (NF-κB) and Mitogen-Activated Protein Kinase (MAPK) signaling pathways, effectively preventing the transcription of pro-inflammatory genes^32^. Consequently, vitamin D significantly alleviates inflammation within adipose tissue.

While prior reports have revealed a link between OP and T2DM, in this study we did not examine the incidence of diabetes in our study population given that whether or not patients were diagnosed with T2DM had no impact on the study. However, individuals with T2DM often exhibit normal to high BMD despite an increased risk of fractures^33,34^. Perturbations in glucose metabolism may impact several bone-derived factors. Hyperglycemia, in particular, can have toxic effects on the differentiation of bone marrow mesenchymal cells (MSCs), leading to a shift towards adipogenesis over osteogenesis^35^. In fact, it has been observed that high glucose levels can activate the non-canonical Wnt/protein kinase C pathway, which may contribute to impaired bone formation and increased adipogenesis^36^ and this pathway upregulates peroxisome proliferator-activated receptor gamma (PPARγ), a key regulator of adipogenesis, which leads to increased adipogenesis and bone loss^37^. Type I collagen is a fibrillary protein, which forms a triple-helix motif and undergoes self-assembly into highly organized fibrils stabilized by enzymatic cross-linking. In addition to natural enzymatic cross-linking, chemical cross-linking may occur between serum sugars and exposed amino acid residues, leading to post-translational modifications of collagen and the production of advanced glycation end-products (AGEs)^38^. The irreversible formation of AGEs through the Maillard reaction or non-enzymatic glycation process has been reported to result in the dysfunction of macromolecules, such as nucleic acids, lipids, and proteins, due to their accumulation^39^. There is increasing evidence to suggest that AGEs, which accumulate with age, can significantly decrease bone density and mineralization^40^. Further research has confirmed that AGEs play a significant role in impaired bone formation by triggering inflammation and bone loss in the pathogenesis of OP^41^.

The primary physiological function of vitamin D is to maintain normal levels of calcium and phosphorus in the body, which is achieved through the activity of its active metabolite, 1α,25-dihydroxyvitamin D [1α,25(OH)2D]^42–44^. Although various dietary factors can affect calcium absorption, such as protein intake, sodium intake, or glucose, the primary regulator is 1,25(OH)2D, which acts through the VDR to synthesize genes and proteins involved in calcium transport^45^. The deletion of VDR results in calcium malabsorption^46^. Vitamin D deficiency can result in secondary hyperparathyroidism and subsequent bone loss, leading to osteoporosis and fractures, as well as mineralization defects that can result in osteocalcin deficiency in the long term. It can also cause muscle weakness, increasing the risk of falls and fractures. Vitamin D status is closely linked to bone mineral density and bone turnover, with the active metabolite 1α,25(OH)2D playing a major role in maintaining calcium and phosphorus balance through the regulation of calcium transport proteins and genes via the VDR, with factors such as protein intake, sodium intake, and glucose levels also impacting calcium absorption. In mice with VDR deletion, calcium malabsorption has been observed^47^.

It should be noted that our study included patients with various comorbidities, including those affecting bone metabolism and blood sugar levels. While we adjusted for these conditions using the CCI, there may still be residual confounding factors that could have influenced the observed associations. Additionally, the presence of specific comorbidities, such as Cushing’s disease or acromegaly, may also influence bone metabolism and blood sugar levels, were not specifically addressed in this study. Future research with a more targeted approach to these specific comorbidities is warranted to further elucidate their impact on the relationship between vitamin D levels and fasting blood glucose.

This study has other limitations. First, this is a cross-sectional study, and it is difficult to complete the investigation of the cause of the association between serum 25(OH)D and FBG. Therefore, this association requires further investigation to determine the mechanism between elevated serum 25(OH)D and decreased FBG. Although some covariates were adjusted for, only the association between serum 25(OH)D and FBG was investigated, and residual confounding variables such as medication effects cannot be ruled out in the analysis. Therefore, further well-designed and stratified cohort studies, with appropriate control groups and accounting for confounding factors, are necessary to better understand the relationship between serum 25(OH)D and FBG.

## Conclusions

We demonstrated a negative relationship between serum 25(OH)D and FBG in the OP population. Another nonlinear relationship and threshold effect were found between the above two variables. The results revealed that the potential effect of vitamin D on blood glucose in patients with OP. We recommend vitamin D supplementation for patients with OP, which can not only improve bone quality in patients with OP, but also improve their blood glucose level. We recommend a serum vitamin D level of at least 23.39 ng/ml for this purpose. However, future prospective intervention studies with a larger sample size are necessary to confirm this hypothesis.

## Ethics

The study protocol was accepted by the ethical committee of Jiangsu University’s affiliated Kunshan Hospital (permission No. 2020-03-046-K01), and it adhered with the Helsinki Declaration. Initially, patient information was documented for the sake of quality improvement at the hospital. Because of the investigation’s anonymous and observational nature, the necessity for informed consent was dropped, and the decision was authorized by the Ethics Council of Kunshan Hospital, Jiangsu University. The identify of the patients was concealed from data analysts.

### Supplementary Information

Supplementary Information 1.

Supplementary Table S1.

## Data Availability

The data that support the findings of this study are available from the corresponding author upon reasonable request.
