# Supplementary material for: Association between serum 25-hydroxyvitamin D and fasting blood glucose in osteoporosis patients
Source: Sci Rep. 2023 Nov 1;13:18812. doi: 10.1038/s41598-023-45504-6 (PMC10620140; doi:10.1038/s41598-023-45504-6)
Supplement: Supplementary file 1 — Supplementary Information 1. [file 41598_2023_45504_MOESM1_ESM.pdf]

**Association between serum 25-hydroxyvitamin D and fasting blood glucose in osteoporosis patients**

**Yao-wei Ye[1,+], Ke Lu[1,+], Yi Yin[1,*], Xu-feng Yang[2], Si-ming Xu[1], Min-zhe Xu[2], Qin Shi[3], Ya-qin Gong[4]**

[1]Department of Orthopedics, the First People's Hospital of Kunshan, Gusu School, Nanjing Medical University, Suzhou, Jiangsu, 215300, China

[2]Department of Orthopedics, Affiliated Kunshan Hospital of Jiangsu University, Suzhou, Jiangsu, 215300, China

[3]Department of Orthopedics, the First Affiliated Hospital of Soochow University, Orthopedic Institute of Soochow University, Suzhou, Jiangsu 215031, China.

[4]Information Department, Affiliated Kunshan Hospital of Jiangsu University, Suzhou, Jiangsu, 215300, China

[+]these authors contributed equally to this work

*** Correspondence:**

Yi Yin

yy-19723@163.com

In the original text, the variable "vitamin D supplementation" was added to Table 1, as presented below:

| *Variables* | *25(OH)D <=20 ng/mL (N) N(%)* | *25(OH)D >20, <=30 ng/mL (N) N(%)* | *25(OH)D >30 ng/mL (N) N(%)* | *P-value* | *P-value** |
|---|---|---|---|---|---|
| *Supplementing Vitamin D categorical* | | | | *0.613* | - |
| *Supplementing Vitamin D* | *114 (10.47%)* | *66 (9.19%)* | *30 (10.83%)* | | |
| *Not Supplementing Vitamin D* | *975 (89.53%)* | *652 (90.81%)* | *247 (89.17%)* | | |

*Result: (N) Mean (SD) Median (Q1-Q3) / N(%)*

And supplementary subgroup analysis examing were conducted, resulting in a stable outcome, as shown below:

| | *N* | *OR (95% CI) P-value* | *P-value for interaction* |
|---|---|---|---|
| *Supplementing Vitamin D categorical* | | | *0.0764* |
| *Supplementing Vitamin D* | *203* | *-0.03 (-0.06, -0.01) 0.0083* | |
| *Not Supplementing Vitamin D* | *1789* | *-0.01 (-0.02, 0.00) 0.0666* | |

[a]*Adjusted for sex, age, BMI, neutrophil count, hemoglobin, albumin, lymphocyte count, high density lipoprotien, category of diagnosis, supplementing vitamin D categorical, season of blood collection, year of blood collection and ICC.*

We propose the following modification to the methods section:

*A total of 2409 consecutive patients with newly diagnosed OP and who were hospitalized were included in the study.*

And we have reconfigured the legend for the inclusion and exclusion criteria, as illustrated below:

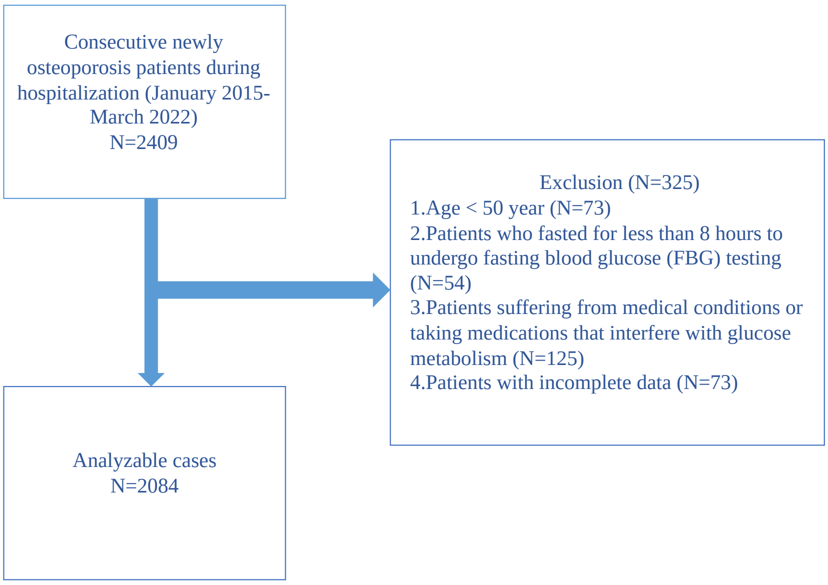

We have added the following paragraph to address this concern in discussion:

*It should be noted that our study included patients with various comorbidities, including those affecting bone metabolism and blood sugar levels. While we adjusted for these conditions using the CCI, there may still be residual confounding factors that could influence the observed associations. Additionally, the presence of specific comorbidities, such as Cushing's disease or acromegaly, may have distinct effects on bone metabolism and blood sugar levels, which were not specifically addressed in this study. Future research with a more targeted approach to these specific comorbidities is warranted to further elucidate their impact on the relationship between vitamin D levels and fasting blood glucose.*

We have added this information to the section on covariates, as follows:

*2.5 Covariates*

*Covariates analyzed in this study were as follows: age; gender; body mass index (BMI); season of blood collection; year of blood collection; neutrophil count (reference range: 1.8-8.3 × $10^9$/L); lymphocyte count (reference range: 1.2-3.8 × $10^9$/L); diagnosis categorical (OP without OPF, OPF); hemoglobin (reference range: male: 130-175 g/L, female: 115-150 g/L); calcium (reference range: 2.11-2.52 mmol/L); albumin (reference range: 40-55 g/L); high-density lipoprotein (HDL) level (reference range: 0.91-1.96 mmol/L); and Charlson comorbidity index (CCI) score (12). All laboratory variables were measured after hospitalization, and patients were asked to fast before blood samples were taken. Comorbidity was assessed using the CCI score.*

We have added the following description below the table 1:

*P-value: ANOVA*

*P-value*: Kruskal-Wallis Rank Test for continuous variables, Fisher Exact for categorical variables with Expected<10*

We have added the following description in statistics:

*P-value carried out with ANOVA, P-value* carried out with Kruskal-Wallis Rank Test for continuous variables or Fisher Exact for categorical variables with Expected<10.*

We have added the following content in the discussion section (regarding the role of vitamin D in regulating fast blood glucose):

*Vitamin D both enhances and promotes insulin secretion from pancreatic β-cells insulin. One of the underlying molecular mechanisms involves the regulation of intracellular Ca2+ concentrations. Non-genomic actions of 1, 25-hydroxyvitamin D3 (1,25[OH]2D3) have been identified as responsible for increasing cytoplasmic Ca2+ levels, leading to activation of insulin exocytosis in pancreatic β-cells and subsequent increased insulin secretion26,27. Vitamin D deficiency can contribute to insulin resistance through several potential mechanisms. For example, the activation of peroxisome proliferator-activated receptor delta (PPAR-δ) by 1,25(OH)2D3 enhances insulin sensitivity28. Additionally, vitamin D improves glucose metabolism by upregulating the Sirtuin 1 (SIRT1)/ insulin receptor substrate1 (IRS1)/ Glucose transporter type 4 (GLUT-4)signaling cascade and enhancing glucose uptake, which is especially evident in high glucose-treated C2C12 mouse myoblast cell (C2C12) myotubes29. Vitamin D also modulates the low-grade chronic inflammation often associated with insulin resistance. Notably, pro-inflammatory cytokines such as Tumor Necrosis Factor-alpha (TNF-α) interfere with peripheral insulin sensitivity by inhibiting insulin-dependent tyrosine phosphorylation of IRS-1. This disrupts the appropriate activation of downstream insulin signaling molecules, including Phosphatidylinositol 3-kinase (PI3K), and the translocation of GLUT-4 to the cell surface30,31. Vitamin D counteracts the release of pro-inflammatory cytokines, such as TNF-α and interleukin 6 (IL-6), and C-reactive protein32. Furthermore, the bioactive form of vitamin D strongly suppresses the activation of the nuclear factor kappa-B (NF-κB) and Mitogen-Activated Protein Kinase (MAPK) signaling pathways, effectively preventing the transcription of pro-inflammatory genes33. Consequently, vitamin D significantly alleviates inflammation within adipose tissue.*
