# Supplementary material for: Association between serum 25-hydroxyvitamin D and fasting blood glucose in osteoporosis patients
Source: Sci Rep. 2023 Nov 1;13:18812. doi: 10.1038/s41598-023-45504-6 (PMC10620140; doi:10.1038/s41598-023-45504-6)
Supplement: Supplementary file 2 — Supplementary Table S1. [file 41598_2023_45504_MOESM2_ESM.docx]

Table S1. Test for interaction of hemoglobin between serum 25(OH)D level and FBG

|  | Hemoglobin <110 g/L | | Hemoglobin >=110 g/L | | interaction |
| --- | --- | --- | --- | --- | --- |
|  | β (95% CI) | *P*-value | β (95% CI) | *P*-value | *P*-value |
| Crude Model^a^ | -0.05 (-0.07, -0.02) | 0.0002 | -0.02 (-0.03, -0.01) | 0.0004 | 0.0280 |
| Model I^b^ | -0.04 (-0.06, -0.01) | 0.0037 | -0.00 (-0.01, 0.01) | 0.3480 | 0.0175 |
| Model II^c^ | -0.04 (-0.06, -0.01) | 0.0063 | -0.01 (-0.02, -0.00) | 0.0099 | 0.0972 |

^a^No adjustment.

^b^Adjusted for sex, age, BMI, neutrophil count, diagnosis categorical, season of blood collection and year of blood collection.

^c^Adjusted for Model I plus hemoglobin, calcium, albumin, high density lipoprotein, lymphocyte count and CCI.

Abbreviations: FBG, fasting blood glucose; β, standard regression coefficient; CI, confidence interval; 25(OH)D, 25-hydroxy vitamin D; BMI, body mass index; CCI, Charlson comorbidity index.
